# Metal/Graphene Composites: A Review on the Simulation of Fabrication and Study of Mechanical Properties

**DOI:** 10.3390/ma16010202

**Published:** 2022-12-26

**Authors:** Julia A. Baimova, Stepan A. Shcherbinin

**Affiliations:** 1 Institute for Metals Superplasticity Problems of the Russian Academy of Sciences, Ufa 450001, Russia; 2 Department of Physics and Technology of Nanomaterials, Bashkir State University, Ufa 450076, Russia; 3 Departement of Theoretical and Applied Mechanics, Peter the Great St. Petersburg Polytechnic University, Polytechnicheskaya 29, St. Petersburg 195251, Russia

**Keywords:** crumpled graphene, metal/graphene composites, mechanical properties, molecular dynamics, strengthening mechanisms

## Abstract

Although carbon materials, particularly graphene and carbon nanotubes, are widely used to reinforce metal matrix composites, understanding the fabrication process and connection between morphology and mechanical properties is still not understood well. This review discusses the relevant literature concerning the simulation of graphene/metal composites and their mechanical properties. This review demonstrates the promising role of simulation of composite fabrication and their properties. Further, results from the revised studies suggest that morphology and fabrication techniques play the most crucial roles in property improvements. The presented results can open up the way for developing new nanocomposites based on the combination of metal and graphene components. It is shown that computer simulation is a possible and practical way to understand the effect of the morphology of graphene reinforcement and strengthening mechanisms.

## 1. Introduction

Metal matrix composites (MMCs) are fundamental structures for the aerospace industry, construction, transportation, and other practical realizations because of their unique properties. The demands for better performances are constantly increasing and one of the essential goals is the search for new composite morphologies with new efficient reinforcement components.

Carbon nano-polymorphs, such as graphene, carbon nanotubes (CNTs), fullerenes, and diamond-like carbon, are well-known for their unique mechanical and physical properties. Two-dimensional (2D) graphene is known as the strongest and thinnest material available and even has better properties than CNTs. In recent years, graphene has been widely used as a reinforcing material in MMC [1,2,3]. Several authors have demonstrated that MMC reinforced by graphene or CNTs shows much better mechanical properties. However, CNTs and graphene flakes can agglomerate during composite fabrication or utilization, which further affects the properties of the composite materials [4].

The most-reported metal matrices that can be reinforced by graphene are Al, Cu, Ni, ferrite, and Ti. Aluminum is well-known as a lightweight metal with good corrosion resistance and formability, as well as multifunctional applications, but with low strength. Copper matrix composites are lightweight, low in cost, have good electrical and thermal conductivities, and are resistant to corrosion. Nickel matrix composites have great corrosion and wear resistance associated with superior resistance to thermal oxidation and have the potential for aero-engine applications. The strengths of Fe and Ti are quite high by themselves; however, they can be further increased by graphene reinforcement. Further, for simplicity, if the special metal is not mentioned, it would be abbreviated as Me. To date, the number of works devoted to the fabrication of graphene/Me or graphene/polyethylene composites are presented [5,6,7,8,9,10,11,12]. Bulk graphene/Me composites can be obtained by different methods, such as the powder metallurgy technique on the basements of different graphene sources, such as thermally exfoliated graphite and reduced graphene oxide) [9]. Al/graphene laminated composites obtained by mechanical ball milling and hot rolling are two times stronger than pure Al [13]. The other way to obtain nanolaminate composites is via the growth of a graphene layer on the surfaces of metal [10].

One of the key factors for mechanical properties is the homogeneous distribution of graphene [9]. The fabrication technique can also considerably affect the final composite structure and it results in better or worse mechanical and physical properties [5,6,7,8,9,10]. The mechanical properties of the composites are defined by the type of graphene pre-cursors [9]. A literature analysis reveals that the current trend is to study the potential of other composite morphology, i.e., crumpled graphene as the matrix and metal nanoparticles as the fillers [14,15,16,17,18,19,20].

Crumpled graphene is one of the most promising and progressive structural materials; it has a high specific surface area, high porosity, and low weight [21,22,23]. Different techniques of the syntheses of three-dimensional (3D) graphene nanostructures based on aerosol preparation from graphene oxide (GO) were used which allows for obtaining the structure with crumpled graphene flakes [24]. Moreover, crumpled graphene can be obtained from graphene using a pre-stretched silicone thin film [25]. It has a wide range of applications, such as supercapacitors [23], hydrogen storage [18,26], and composites [9], to name a few. Such structures showed to be stable in comparison with single-layer graphene and can be obtained more effectively. They can successfully reinforce graphene/metal composites.

**Figure 1:**
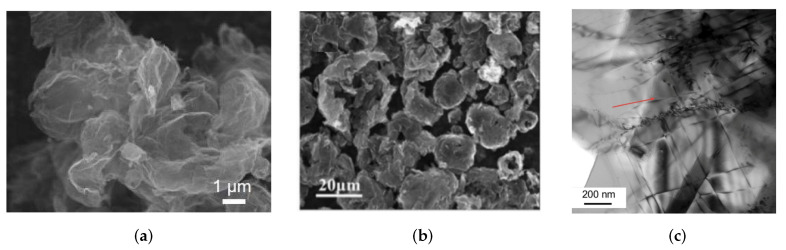
(**a**) SEM images of crumpled and spherical rGO powders. Reprinted with permission from [21]. (**b**) SEM image of crumpled graphene balls. Reprinted with permission from [22]. (**c**) Bright-field image of Al/graphene composite. Reprinted with permission from [27].

Figure 1 shows the example of the crumpled rGO powder (a), crumpled graphene balls (b), and Al/graphene composite (c). Graphene papers shown in Figure 1a are obtained from crumpled reduced graphene oxide (rGO) spheres. It is shown that graphene papers with tunable inner pore structures can be fabricated from crumpled graphene and ultrasonication is useful for tailoring the resulting morphology [21]. In Figure 1c, the example of the Al/graphene composite is presented. To date, fabrications of such composites are already realized [27].

The complexity of the composite structures and the difference in their morphology cause significant difficulties in an experimental indication of the relationships between structure and properties or external treatment and properties. The understanding of the strengthening mechanisms by graphene reinforcement is also quite complicated directly from the experiment. The molecular dynamics (MD) simulation is a suitable tool used to investigate the properties of the nanocarbon-reinforced MMCs and allows the consideration of crystal orientations, interfacial structures, and mechanical treatment in a wide range of working temperatures and strain rates, etc.

Several reviews concerning the metal/graphene composites from different points of view have been published [2,3,16,28,29], but there is a lack of comprehensive summaries of simulation techniques that allow for the simulation fabrication and study of the properties of such composites. This work is a reliable review of the newest scientific papers focusing on the studies of graphene/metal composites. The state of research on their mechanical properties is summarized. This review analyzes recently developed graphene/metal composite materials and their properties and the utilization of MD as an efficient tool for studying such structures.

## 2. Graphene/Metal Composites

### 2.1. Morphology of Graphene/Metal Composites

**Figure 2:**
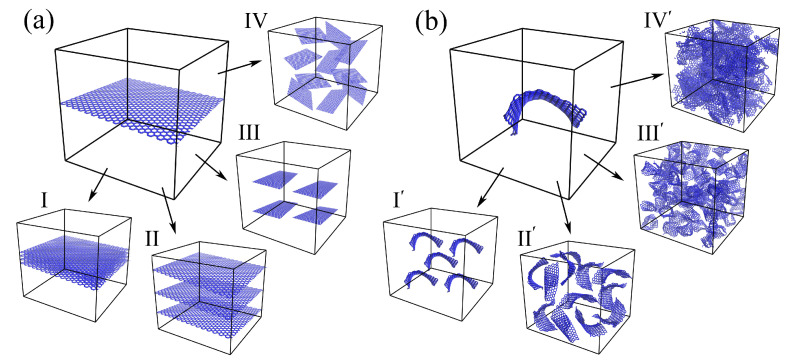
Schematic of metal/graphene composites with (**a**) planar graphene and (**b**) crumpled graphene. Metal atoms are not shown, graphene is shown in the blue color.

To date, mainly the MMC composites reinforced by graphene are considered by MD. The simplest morphology is a metal matrix with one graphene layer inside, which allows for studying different mechanisms of the composite formations, mechanical behaviors, etc. In Figure 2, the schematic representation of the composite morphology is presented. Here, metal atoms are not shown, but in the model, the empty space is filled with metal atoms. it means that graphene is embedded in the metal matrix. The morphology of the composites based on graphene and metal matrix can be very different depending on the shape of graphene flakes and the amount of graphene in the metal matrix. The properties of the composites will considerably depend on the graphene distribution, shape, size, and other different parameters that define the whole morphology.

As can be seen from Figure 2, two different precursor structures can be considered. The idea to reinforce the metal matrix by the graphene plane arose in 2009 and showed great success [28]. The mechanical properties of such composites increase considerably, which opens new opportunities to create different morphologies with graphene flakes of different distributions and different orientations. The presented morphologies are just examples and the sizes and shapes of graphene reinforcements are shown randomly. Four examples are presented in Figure 2a: I, II—several graphene layers with different interlayer distances, III—several small flakes, monotonously distributed in a metal matrix, IV—several metal flakes randomly distributed in the metal matrix. Here, for simplicity, small graphene layers will be called graphene flakes and large-area graphene will be called graphene.

For such composites, many different factors affecting the mechanical properties of the composites are distinguished: the size of graphene flakes, their orientation (zigzag, armchair, or chiral), the number of graphene flakes inside the metal matrix, the distance between graphene sheets. Moreover, planar single-layer graphene is rarely used for MMCs fabrication since it is unstable in the planar form. The graphene tends to agglomerate and stack to multilayered flakes or even to graphene networks [28].

Thus, it was realized that, inside, the metal matrix graphene in its equilibrium state is not planar and can be transformed into a crumpled/rippled state during the fabrication of the composite, especially if the metal matrix is melted. It is much more natural to consider the system of crumpled graphene flakes instead of planar graphene. As can be seen from Figure 2b, again, graphene can be large areas or small flakes. Studying different distributions of crumpled graphene in the metal matrix, the important conclusion was that graphene flakes can be connected to the whole graphene network and reproduce such structures as crumpled graphene or graphene aerogel. Such a graphene network can show even better strength in comparison with the separated flakes.

**Figure 3:**
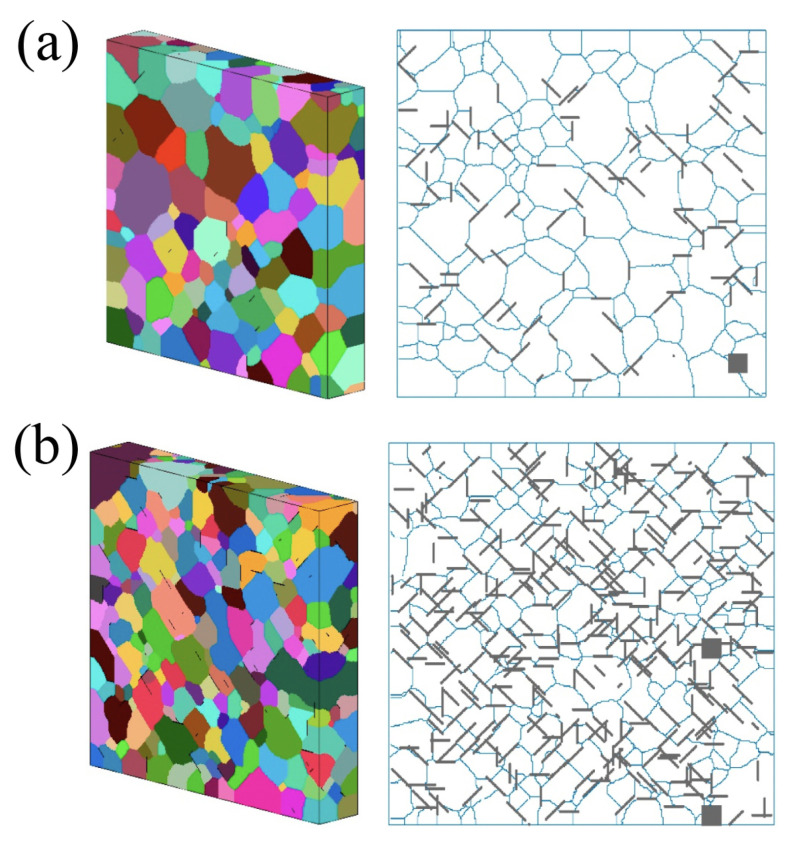
The 3D and 2D cross-sectional microstructures of the Al MMC with the distribution of graphene flakes: (**a**) 1 wt.% and 15 μm of graphene, (**b**) 4.5 wt.% and 15 μm of graphene. Reprinted with permission from [30].

In real experiments, it is quite complicated to obtain the monocrystal metal matrix. Commonly, there is a polycrystal with different grain sizes. This is one of the main contributing morphology factors affecting the mechanical and physical properties of the composite. Thus, graphene flakes can also be distributed differently and considerably affect the grain size and resulting mechanical properties [30]. In Figure 3, one can see the 3D and 2D cross-sectional microstructures of the Al MMC with the distribution of graphene flakes of 15 μm with 1 wt.% (a) and 4.5 wt.% (b) concentrations. Further, the effects of different presented morphologies on the mechanical properties and strengths of the composites will be considered.

The same was shown for CNTs, which have a strong pinning effect on grain boundaries, which results in a low ductility of the composites [31,32]. In the present review, even though we only describe the composites based on graphene and metal, such carbon polymorphs as graphene and CNTs have very similar properties and effects on the strength of MMC. It is known that both polymorphs have high strengths by themselves, although it depends on the structural peculiarities and fabrication techniques. The first works on carbon/Me composites were dedicated to the MMC reinforced by CNTs, then, since graphene was experimentally exfoliated, the scientific community moved toward MMC reinforced by graphene. Despite the resulting composites having some differences in physical and mechanical properties, there are a lot of similarities with the common mechanisms of strengthening for MMC with CNTs and graphene.

### 2.2. Interaction between Graphene and Metal

The deposition of C atoms on different metal sources, along with the diffusion of carbon atoms, and further segregation on the surface were extensively studied both by the experiment and simulation. Commonly, such studies are conducted to analyze the process of graphene or CNT growth, but in terms of composites, more important characteristics can be obtained from such studies pertaining to how actively graphene and metal can interact with each other. Such simulations are often conducted by *ab initio* (or first principles), semi-empirical and empirical methods, based on when the data from the experiments are used to determine the parameters of the system. Thus, the obtained results can be further used to understand the MD results or to prepare the parameters of interatomic potential for MD simulations.

Different metals have different adhesion energy, solubility, and catalytic properties in contact with carbon [29]. For example, a linear dispersion at the Dirac point was observed for Cu, Ag, and Au, which means that electronic decoupling between the metal and the graphene took place. The distance between the metal and graphene is close to 3 Å, and this value is characteristic of the van der Waals (vdW) interaction. On the other hand, Co, Ni, Ru, Rh, and Re show stronger interaction and shorter distances [29,33].

It was shown that graphene demonstrates strong bonding with metals, such as Ti and Ni due to the coupling between open *d*-orbitals, but weakly interacts with Cu [34,35,36,37,38], which allows using the Lennard–Jones (LJ) or Morse potential for C–Me interactions [39,40]. Among various metals, Al, Cu, Ag, Au, and Pt have weak cohesion with graphene, while Co, Ni, and Pd have strong cohesion [34,41,42,43,44,45]. The *ab initio* simulations suggested that the Me/graphene interaction is vdW, rather than a chemical bonding interaction [46]. Although the interaction energy for Ni is high and for Cu is low, both metals are used in graphene epitaxial growth, nanoelectronics, and functional composite applications. However, it is more difficult to form a stable Cu/C compound [47,48], than, for example, Ti/C or Ni/C.

It should be noted that the results for the interaction between single or coupled C atoms and the metal surface can differ from the results describing the interaction between the graphene layer and metal surface [17,29,49]. Even the results, obtained by different methods differ considerably. It was revealed in [29] that there is a wide range of interaction energies between Ni and graphene, obtained by different potential functions, although all predict a stable configuration. For Ni/graphene, cohesion energy was found to be different in the experiment—6.76 J/m${}^{2}$ [50] and 72.7 J/m${}^{2}$ [51], compared with first principle calculations—1.64 J/m${}^{2}$ [35]. The adhesion energies of graphene on the Cu substrate calculated in the experiment were 0.72 J/m${}^{2}$ [52] and 12.75 J/m${}^{2}$ [51]; calculated by the first principle, it was 0.40 J/m${}^{2}$ [35]. Different parameters for the Morse potential should be used for different positionings of Ni atoms on the graphene surfaces [53,54]. Thus, to obtain the realistic parameters for the potential of the carbon–obtain potential parameters for the description of the interaction between a metal nanoparticle and graphene flake, it is better to consider a curved graphene surface (fullerene) interacting with metal nanoparticle [49]. The specific energies of metal/graphene interfaces are very important and affect the resulting strength of the composite [55].

### 2.3. Mixing of Graphene and Metal Nanoparticles

To date, different methods were employed to obtain different kinds of graphene hybrids—a mixture of graphene and Me or Si nanoparticles, graphene in a metal matrix, and the metal nanoparticles wrapped by graphene, to name a few. The morphologies of such hybrid structures can be very different depending on the fabrication technique and materials–precursors. One of the main objects of this review is the graphene/metal composites based on a mixture of graphene flakes and metal nanoparticles. Graphene/metal nanoparticle hybrid systems were already obtained for noble metal nanoparticles (Au, Ag, Pt, and Pd) [56,57] and the transition metal nanoparticles (Ni, Co, Cu) [58]. Among the different methods of fabrication of the hybrid graphene/Me systems are the direct growth of the nanoparticles on the graphene surface and solution mixing—the mixture of graphene flakes and pre-synthesized nanoparticles. In both approaches, system units can be bonded either by chemical bonding or non-covalent interactions. Despite various strategies for mixing nanoparticles and graphene precursors being developed to date, a lot of unsolved issues remained: how to better assemble nanoparticles on graphene, how to make controllable morphology, the suitable density of the composite, and improved properties following the practical requirements.

A new way to fabricate the composite with controllable morphology where graphene wrapped the nanoparticles has been reported in [59]. The method of colloidal coagulation can be used to obtain a relatively uniform distribution of nanoparticles of the desired size wrapped by graphene. Similarly wrapped structures were also obtained by mixing graphene oxides and different nanoparticles further combined into a single structure in the solution [60,61]. Catalytic syntheses of the graphene with metal nanoparticles were also studied to understand the effect of different reaction parameters, such as the catalyst concentration, reaction temperature, and reaction time on the fabrication of graphene with metallic nanoparticles [56,62]. It is interesting that the increase in the shear of metal atoms increases the number of nanoparticles and the specific surface area of graphene.

One of the well-known methods is the mechanical mixing of graphene and various nanoparticles. Composites based on graphene oxide and nanoparticles were successfully prepared through a simple ball-milling method for different applications [63,64]. However, this method should be applied carefully since it is quite hard to achieve uniformity in the structure. Moreover, the crystal structure of the components can be destroyed during ball milling [65]. The mixed structure can be further pressed and sintered to obtain a dense composite structure [66], but the obtained structure again can be quite irregular with non-controllable properties. A good review of the fabrication of graphene/Mg composites is provided in [65], where different composite formation methods are described.

## 3. Molecular Dynamics Simulation

Molecular dynamics simulation is based on the mathematical description of the interaction between atoms. The accuracy of predictions made based on the simulation results depends on the accuracy of this description and application to a specific problem. Classical methods of interaction are described using the potential function $U({\vec{r}}_{1},{\vec{r}}_{2},\ldots ,{\vec{r}}_{N})$, which determines the potential energy of a system of *N* atoms as the function of their coordinates. The forces acting on each atom are calculated from this potential function:

$${\vec{F}}_{i}=-\frac{\partial U({\vec{r}}_{1},{\vec{r}}_{2},\ldots ,{\vec{r}}_{N})}{\partial {\vec{r}}_{i}}\equiv -\nabla_{i}U\left({\vec{r}}_{1},{\vec{r}}_{2},\ldots ,{\vec{r}}_{N}\right).$$

The simplest form of the description of the interatomic interaction is the pair potential. Strictly speaking, this potential does not have quantum mechanical justification. However, due to its simplicity, it is often used in modeling. At the approximation of pair potentials, the energy of a system of particles is represented as the sum of potential energy interactions of all pairs of atoms:

$$U\left({\vec{r}}_{1},{\vec{r}}_{2},\ldots ,{\vec{r}}_{N}\right)=\frac{1}{2}\sum_{i=1}^{N}\sum_{j=1,(j\neq i)}^{N}\varphi \left(r_{i,j}\right),$$

where $r_{i,j}=\left|r_{j}-r_{i}\right|$ is the distance between pair of atoms. The most common pair potentials are the Lennard–Jones and Morse potentials.

For the structure, where two types of atoms are considered, it is necessary to choose a potential that will take into account three types of interactions. For graphene/metal composites, the potential should include covalent bonds for graphene, interactions of the metal atoms with graphene, and interactions between metal atoms inside the metal part. Thus, the potential function for the composite can be defined as the sum of three potential energies of carbon–carbon $U_{C-C}$, carbon–metal $U_{C-Me}$, and metal–metal $U_{Me-Me}$ interactions correspondingly:

(1) $$U_{system}=U_{C-C}+U_{C-Me}+U_{Me-Me}$$

The interaction of C-–Me is at least one order of magnitude smaller than the interactions between Me atoms [67], while covalent bonding in the basal plane of graphene is even stronger. Usually, the first term ($U_{C-C}$) is calculated using the AIREBO potential to describe the interatomic interactions between carbon atoms [68]:

(2) $$U_{C-C}=\frac{1}{2}\sum_{i}\sum_{i\neq j}\left[U_{ij}^{REBO}+U_{ij}^{LJ}+\sum_{k\neq i,j}\sum_{l\neq i,j,k}U_{kijl}^{TORSION}\right],$$

where $U_{ij}^{REBO}$ is the hydrocarbon REBO potential developed in [69], $U_{ij}^{LJ}$ term adds longer-ranged interactions using a form similar to the standard LJ potential, and $U_{kijl}^{TORSION}$ describes various dihedral angle preferences in hydrocarbon configurations. This potential is very famous for the study of different carbon structures and their properties [17,18,28].

Understanding how the simulation process itself affects the results is also important for such a complex system. In [70], it was shown that the loading of graphene in a dynamic regime results in brittle behavior instead of ductile or needs better structure relaxation. In [19], the mechanical properties of crumpled graphene with Ni nanoparticles inside are studied under dynamic and incremental loading. It was shown that even the strength or fracture strain of graphene can be very different at these loading techniques. The tension strain rate affects the resulting ultimate tensile strength by decreasing the strain rate and decreasing the critical stress and strain. If the tension at zero and room temperature are considered, the ultimate tensile strength will be lower at 0 K, and close to the theoretical strength. The deformation behavior of composite is very similar and does not crucially depend on the deformation techniques (dynamic and incremental loading). However, the direction of the uniaxial tension using dynamic loading will affect the composite strength [18].

The other important factor is interatomic potential. The interaction between metal atoms and carbon atoms can be represented by pairing the interatomic potential—LJ or Morse. As it was mentioned before, graphene has strong bonding with Ti and Ni and interacts weakly with Cu [34,35] and Al [34,41,42,43,44,45]. Thus, the LJ potential can be effectively used for such interactions [36,37,38,39,40]. However, the charge transfer, which is part of the attraction forces and results in their quick decay, and is also a covalent interaction between Ni and C atoms [71,72], cannot be reproduced by LJ potential. Thus, for Ni or Ti, another interatomic potential should be taken into account which would be discussed further. It was shown that the Morse potential has been successfully used to describe the interaction of metals with graphene and silicene [73].

Since the 1980s, several methods have been proposed to describe interatomic interactions in metals. It is quite important to take into account the complex nature of the interaction of atoms. Thus, for metals, potentials are built from quantum theory and the electronic structure of crystals, which is conducted based on first principles (*ab initio*). Some of these methods should be named: the embedded atom method (EAM), effective medium theory, Finnis–Sinclair potential, etc. The Morse potential can also be applied to study various properties of metals.

Further different interatomic potentials for the simulation of graphene/metal systems would be considered.

### 3.1. Interatomic Potentials

#### 3.1.1. Lennard–Jones Potential

The Lennard–Jones (LJ) potential can be described as

$$U\left(r_{ij}\right)=4\epsilon \left[{\left(\frac{\alpha }{r_{ij}}\right)}^{12}-{\left(\frac{\sigma }{r_{ij}}\right)}^{6}\right],$$

where α and ϵ are constants with dimensions of length and energy, respectively. The distance between atoms at which this potential reaches the minimum value is equal to $r_{min}=1.22\alpha$, and the minimum value of the potential is $U_{min}=-\epsilon$. The first part in the brackets (12th power) describes repulsion at a close range. The second part (6th power) simulates attraction at long distances, associated with the dipole–dipole interaction. The coefficients ϵ and α are determined by fitting the binding energy of the metal to the equilibrium distance between atoms.

For metal/graphene composites, the LJ potential is most commonly used for the simulation of the Me–C interaction. It is applied to both strong and weak interactions with graphene metals. As it was shown, nonbonded Me–C interactions can be successfully reproduced by the LJ potential [7,8,36,39,46,54,70,74,75,76,77,78,79,80,81,82,83,84,85,86,87,88,89,90,91,92,93,94,95,96,97,98,99,100,101,102,103,104,105,106,107,108,109,110,111,112,113,114,115]. The LJ potential reflects the interaction between carbon and metal with very high accuracy, especially for such issues as the effects of chirality and interlayer thickness and graphene rippling on the mechanical properties and dislocation dynamics in graphene/Cu [8,79,81,85,89,92,93,94,102,103,109], graphene/Al [111], graphene/Ni [114], graphene/Al [106,110,112] composites. As can be seen, a commonly simple LJ potential is used for the simulation of the graphene/Cu or graphene/Al system, rather than for the graphene/Ni system. The syntheses and growth of graphene on metal substrates are also well reproduced by LJ potentials [100,104] as well as thermal conductance through graphene/metal interfaces [39,75,80].

#### 3.1.2. Morse Potential

The Morse potential can be written as

(3) $$U^{Ni-C}\left(r\right)=D_{e}\left[{\left(1-e^{-\beta (r-R_{e})}\right)}^{2}-1\right],$$

where $D_{e}$ is the binding energy, $R_{e}$—distance for potential energy minimum, and β—potential parameter.

Three parameters $D_{e}$, $R_{e}$, and β, allow one to adjust potential, in addition to the binding energy and lattice parameter, to the bulk modulus *B* of the crystal associated with the slope of the potential near its minimum. This potential is also often used to model metals with f.c.c. and h.p.c. lattices.

One of the main things that should be reproduced by the potential is the bonding stiffness/hardness for different metals. Such things are usually studied during the simulation of catalytic syntheses of graphene or CNTs [29,116], the growth of carbon on the metal surface [117], and the interaction between fullerene or small graphene flakes with metal clusters [49,73]. It was shown that the Morse potential can successfully reproduce such an interaction if the parameters are calibrated using experimental or abinitio techniques. Moreover, this potential was also used for the simulation of graphene/metal composites [16,17,18,19,37,48,117,118,119,120,121,122,123,124,125,126].

It was shown in [127] that the Morse potential provides a more precise and generalized description for modeling covalent materials and surface interactions. This potential was used both for the simulation of the fabrication process as well as for the study of mechanical and physical properties [16,19,20,128].

#### 3.1.3. Other Potentials

The ‘many-body’ character of the interaction between carbon and such metals as Ni or Ti requires more complex potentials for different problems.

For example, a more general bond order method was designed and called ReaxFF potential for hydrocarbons on Ni [129,130,131,132,133]. ReaxFF is a reactive force field that can be used to study the mechanical properties of graphene and metal/graphene composites taking into account both covalent bond breaking and nonbonded interactions. It was confirmed that the ReaxFF force field is suitable for reproducing the mechanical properties of graphene and its interaction with a metal surface [134,135,136]. In [136], ReaxFF was used to study the interaction of planar and wrinkled graphene and Cu surface. It was confirmed that ReaxFF could provide useful information for interactions of graphene with metal surfaces, indicating its further application to battery current collector/electrode interface simulations. In [137] from the comparison of Morse and ReaxFF potential, it was shown that both could reproduce the same structural state of graphene interacting with Ni nanoparticles. Qualitatively the same results were obtained by both Morse and ReaxFF potentials, but the calculation time is considerably increased with the ReaxFF. More complex ReaxFF potential should be used to study small systems where the carrier distribution is of high importance and for short-time processes. Moreover, in [138], various potentials for graphene were analyzed against *ab initio* data and it was found that the ReaxFF potential performed poorly.

In [139], a force-field-based MD simulation was used to study the new type of composite composed of CNTs and Cu nanoparticles. It is an *ab initio* force field and it has been proven to be applicable in the investigation of the mechanical properties of the CNT contact with Cu.

The other type of interatomic potential for the simulation of Cu/graphene structures is the charge-optimized many-body potential (COMB) [140,141]. In [140,141], the third generation of the COMB potential (COMB3) was developed. The COMB3 formalism has already been successfully used to study hydrocarbons on Cu surfaces [140] as well as surface oxidation of Cu [142]. Moreover, this potential allows for studying more complex structures with H and O atoms, which allows the simulation of graphene on the Cu surface [143]. The ReaxFF and COMB potentials have been built around the same two fundamental concepts of self-consistent charge equilibrations and bond orders [141].

## 4. Results and Discussion

### 4.1. Simulation of the Composite Fabrication

Recent progress in computer science has led to an increase in the simulation studies of composite fabrication. MD simulation is a good tool used to study and predict this non-simple process, for example, fabrication mechanisms, structures, and dynamics of surfaces and interfaces. During MD simulations, such processes can be described in detail since various factors can be easily considered: (i) process temperature; (ii) type of the reinforcement and metal matrix; (iii) deformation treatment; (iv) variety of structures.

#### 4.1.1. Deposition of Graphene on the Metal Surface

The process of deposition of graphene on the metal surface is one of the most studied by different simulation techniques. It is not exactly the fabrication process but it is very important for understanding how graphene can be introduced to the metal matrix. The interaction of metal nanoparticles with the graphene surface has also been widely studied over the past several decades [144]. The bonding process is strongly connected with the different types of interaction between carbon and different metals, as mentioned in Section 2.2. For example, the growth of graphene on Cu [98] is a surface-mediated process and took place on the pre-melted surface [145]. While for Ni with higher interaction energy, the process of graphene growth is much simpler [51].

**Figure 4:**
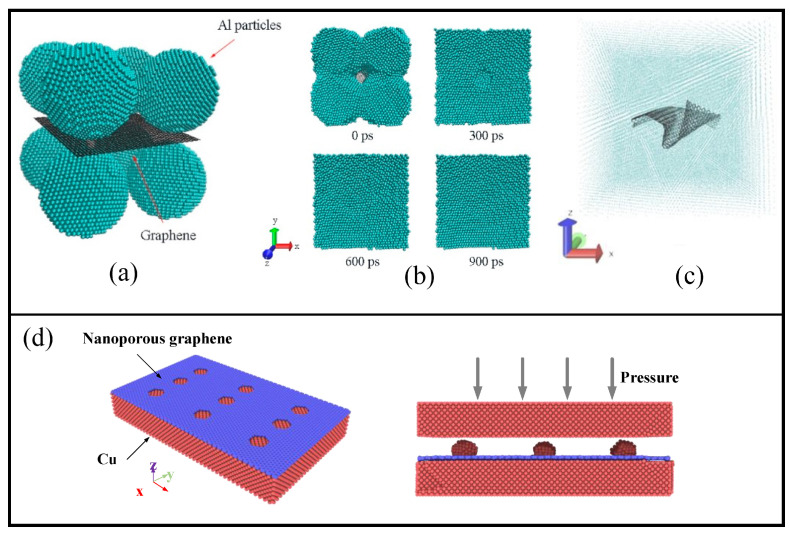
(**a**) Al–graphene system. (**b**) Atomic configurations of the composite systems at different sintering times. Reprinted with permission from [128]. (**c**) Graphene in the metal matrix after sintering. Reprinted with permission from [14]. (**d**) Atomic configurations of the Cu-nanoporous graphene composite model. Reprinted with permission from [13].

With many metals, graphene interacts by weak vdW forces. Since this interaction can be easily destroyed even at room temperature, it reduces the strength of such layered composites. The weak vdW contact between the graphene and metal matrix significantly reduces the mechanical performance of such composites. In [13], a new bonding method with a low bonding temperature and good dependability is used to obtain Cu/graphene. In Figure 4d, an example of the initial structure consisting of a Cu layer and nanoporous graphene is presented. At first, nanoporous graphene is deposited on the Cu surface, and then C atoms are deposited over graphene and segregated into Cu islands. After that, another Cu layer is deposited to obtain a layered structure. The presence of such Cu islands leads to the increase of the interfacial shear strength between graphene and Cu surface. The effect of such a new methodology on the strength of the composites will be discussed further.

#### 4.1.2. Graphene as the Reinforcement

The other effective way to obtain the composite is compression of the MMC reinforced by graphene. In [89], models of pure Cu, with one graphene layer inside and different graphene layers under compression are considered. During compression, a lot of dislocations and other defects appear in the structure which leads to an increase in the composite strength. The main reason for the strength increase is the interaction between dislocations and graphene layers. However, at some tensile strain, this developed dislocation structure in combination with graphene layers leads to the fracture of the composite.

Powder metallurgy is one of the most important techniques for processing composites at low temperatures with the ability to incorporate high-volume fractions of reinforcements. However, the work of reinforcing phases will be affected by the various dispersing technologies such as ultrasonic dispersion or ball-milling. As it was shown in [66], the structure of the composite obtained from Mg, Zr, and graphene powders by cold pressure followed by sintering showed an irregular distribution with GFs with wrinkled edges. However, especially uniform distribution of GFs in metal matrices can improve the mechanical and corrosion properties of the composite. Thus, understanding the compression, sintering process, and densification is of high importance. Mechanical properties of the graphene/Me composites under different loading including compression have been studied previously by different simulation methods [146,147,148]. The simulation of such experimental techniques can show, in detail, the densification of the structure. For example, sintering results in a porosity decrease, volume shrinkage, and density increase. Even the sintering process by itself can speed up the metal reinforcing by graphene.

In [147], the Al/graphene composite was studied under compression. It was shown that compressive strength behavior and elastic modulus are considerably dependent on the initial morphology of the structure. Under compression, the density of the composite increases with an increase in the reinforcement share. Especially graphene addition could lead to a faster density increase in comparison with other carbon polymorphs. In [128], a model for powder sintering of Al nanoparticles with a graphene layer inside is studied by molecular dynamics simulation. As can be seen from Figure 4a, the initial structure consisted of eight spherical Al nanoparticles and a square nanoplatelet of graphene in the center of the simulation cell. Interaction between Al atoms is described by the EAM potential, bonding inside graphene—by the Tersoff potential, and Al–C interaction—by Morse potential. The sintering process was visualized to monitor the changes in the surface morphologies of the composite (see Figure 4b). It was revealed that graphene is more favorable for sintering in comparison with the diamond nanoparticles since densification becomes faster with no pores appearing near the reinforcing element.

It was found [14,15] that the tensile load of the composite can be realized through graphene bending. Such a crumpled structure is shown in Figure 4c, where the composite is presented after sintering with the single-layer graphene inside the metal matrix. Thus, graphene may considerably improve the mechanical performance of the reinforced composite, but it is impossible to approach the level of the strength of graphene itself.

#### 4.1.3. Graphene Network as the Basement for the Composite

From this point of view, the idea to create a graphene network in the metal matrix is arise. Such a network can give much more strength to MMC. It was realized in [20], where the formation of the strong graphene network during plasma sintering was studied by MD for graphene/Ni composites. From the composite, obtained by sintering with the graphene layer as the reinforcement composite, we move toward a more complex architecture, where reinforcement is the complex graphene network. Ni nanoparticles are wrapped by graphene flakes, which are bonded during the sintering process. It was shown that the diffusion of Ni through the graphene network took place, which is very important for the densification of the composite. Diffusion of Ni is also allowed by the thermal mismatch between Ni and graphene lattices. The same results and mechanisms were observed experimentally.

The other effective way of fabrication of composite with a graphene network is the pressure-temperature treatment, presented in [16,19] for graphene/Ni composite. Different external and internal factors affect the composite strength, namely: (1) the size of metal nanoparticles; (2) the temperature of hydrostatic compression; (3) the application of annealing. The main factors that affect composite fabrication are the nanoparticle size, the orientation of the structural units, and the temperature of the fabrication process. The temperature of compression is very important: on the one hand, it should be about 0.6–0.7$T_{m}$ ($T_{m}$ is melting temperature), because metal nanoparticles should not be melted during densification. If nanoparticles would be melted, separated atoms will spread over graphene flakes and will almost not affect the composite properties. On the other hand, compression temperature should be big enough (commonly, bigger than 600 K) to allow the bond destruction in the basal plane of GFs to facilitate the bonding between neighboring flakes. Better mechanical properties also can be achieved for the Ni/graphene composite after annealing at 2000 K.

**Figure 5:**
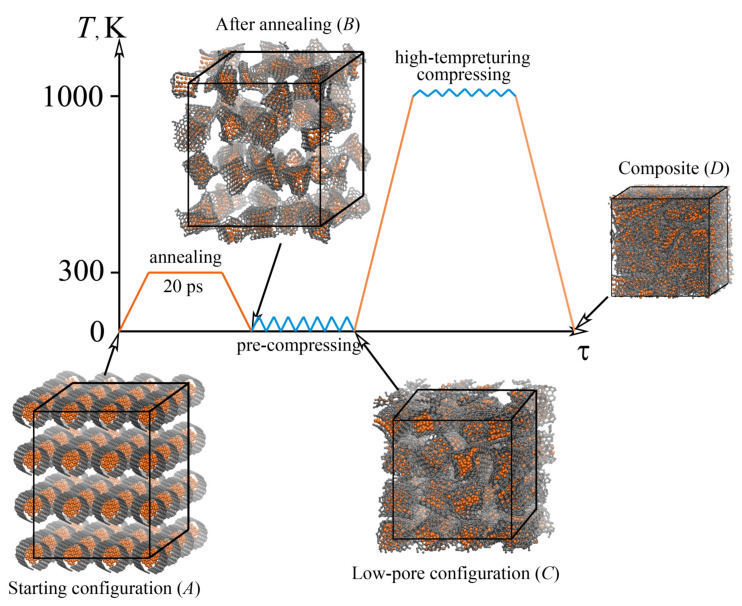
Scheme of the performed tests and their corresponding conditions to obtain a Ni/graphene composite. Snapshots of the structures in the initial (A), final (D) states, and states after each processing stage (stage B, after annealing at 300 K for 20 ps; stage C, after pre-compression). Nickel atoms are shown in orange and Carbon atoms—in gray color. Reprinted with permission from [19].

Moreover, the fabrication scheme was developed, as shown in Figure 5. From the snapshots of the structures in the initial (A), final (D) states, and states after each processing stage (stage B, after annealing at 300 K for 20 ps; stage C, after pre-compression) it can be seen that the initial structure preparation can considerably affect the resulting structure. If the temperature of 300 K is considered, the process cannot be called annealing, but it would be just exposure. However, this fabrication technique can include also pre-annealing, for example at temperatures from 1000 to 2000 K.

### 4.2. Mechanical Properties

To study the mechanical behavior, tensile deformation can be applied which even allows for comparison of the experiments and simulation results. The mechanical properties and deformation behaviors of different graphene/Me composites can be obtained under different conditions. MD has always been used as an effective way to study graphene coatings under nanoindentation [79,114,149,150] or scratching [151,152]. MD simulations of graphene/Me composites can shed the light on the connection between structure peculiarities and properties, understand the deformation and strengthening mechanisms, find a better way to reinforce the composite, etc. It is known that the mechanical properties of graphene are anisotropic which can result in the anisotropy of the mechanical behavior of the composite. Thus, the tensile mechanical deformations considerably depend on the tension direction.

Let us consider the simple laminated structure first: the graphene layer between two layers of metal (see Figure 2a).

The next step for further property improvement is to add more graphene layers to the metal matrix. It can be done variously: (i) to add bi- or multi-layer graphene (see Figure 2a, I); (ii) to add several graphene layers of the same size as the metal matrix with the distance between layers, which can be varied (see Figure 2a, II); (iii) to add several small graphene layers (see Figure 2a, III). In all of these cases, graphene anisotropy will considerably affect the strength of the composite. If tension is applied along the graphene layer, the strength will increase, while if it is applied normally to the graphene layer, graphene will easily separate from the metal surface [153]. However, in [13], the new method to overcome this weakness is discussed. It is shown that if nanoporous graphene is used, the bonding between Cu and graphene can be considerably increased.

As mentioned previously, it is better to consider polycrystalline samples since the interaction between graphene and grain boundaries can significantly affect the resulting mechanical properties. If the crystallization of metal is considered, the content of graphene can affect the uniformity of grain distribution and the final grain size of MMCs [30]. Moreover, it was shown that the graphene added to the metal matrix tends to unite, twist, and shift toward the grain boundaries [154]. However, the high content of graphene can result in its agglomeration and the appearance of defects, which will reduce its mechanical properties. The size and number of graphene layers are very important: large graphene flakes can contact and transform into a graphene network; however, it would increase the strength of monocrystals, but can decrease the strength of polycrystals, which depends on the grain size.

#### 4.2.1. Cu/Graphene Composites

**Figure 6:**
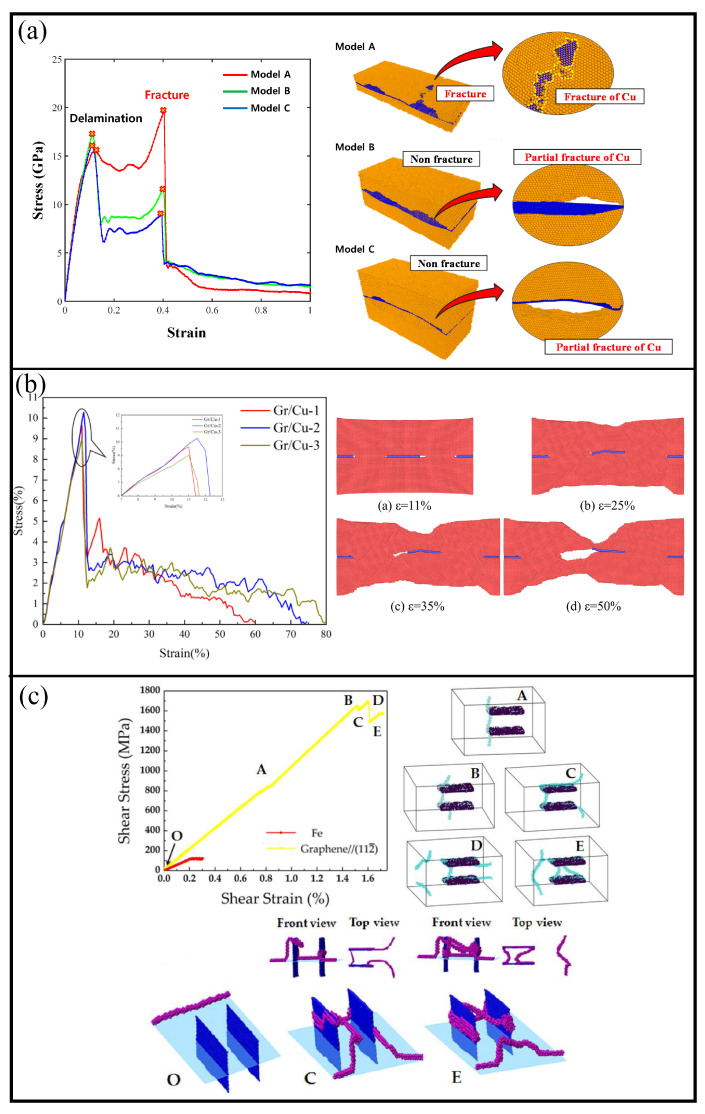
(**a**) Stress–strain curves for Cu/graphene composite during uniaxial tension along the zigzag direction of graphene with the snapshots of the structure at the fracture point. Three different thicknesses of the Cu part are considered: Model A (4.8 nm), Model B (9.8 nm), and Model C (14.3 nm). Reprinted with permission from [153]. (**b**) Stress–strain curves during tension for structures with different coverage and arrangement of graphene (Gr/Cu-1, Gr/Cu-2, and Gr/Cu-3, respectively). The snapshots of the Gr/Cu-1 composite during tension are presented. Reprinted with permission from [155]. (**c**) Stress–strain curves of the pure iron and the graphene/Fe composite. A pair of single-layer graphene nanoribbons at different locations in the metal matrix is used as the reinforcement. Reprinted with permission from [156].

In Figure 6, an example of the mechanical behavior of the Cu/graphene composite is presented during tension along the zigzag direction of the graphene layer [153]. The difference between Model A, B, and C is the thickness of the Me layer.

Composite with the lowest Cu thickness has the lowest stress value among the composites at the time of delamination (first critical point). The greater the thickness of the Cu layer, the lower the fracture strength. From the snapshots of the structure, it can be seen that for the Cu layer with small thicknesses, fracture took place in a metal part rather than on graphene. For the structure with the average and maximal thickness, the graphene sheet was broken earlier than Cu completely fractured. However, for tension along the armchair direction, slightly different results were obtained: composite with the maximal Cu thickness has the lowest stress value among others at the time of delamination initiation, while the fracture behavior is the same as during tension along zigzag. It was concluded that an increase in Cu thickness reduces the strength of the graphene/Cu composites.

In Figure 6, stress–strain curves during tension are presented for structures with different coverage and arrangements of graphene (Gr/Cu-1, Gr/Cu-2, and Gr/Cu-3, respectively) [155]. During plastic deformation, the Cu matrix slides along graphene and a cavity starts to grow at the interface. For the structure with a bigger graphene layer (Gr/Cu-2) or bigger graphene content, strength is higher since the dislocation density is higher. For multi-layer graphene (Gr/Cu-3), the voices are larger than for single-layer which leads to a decrease in strength. In common, the strength of laminated metal/graphene composites is strongly connected with the lateral size of graphene layers [55].

For a better understanding of the strengthening of the metal by graphene, it is interesting to consider nanoindentation of the surface covered by graphene. In [150], it was shown that graphene changes the slip behavior of dislocations in Cu. Parallel to the interface slip of dislocations took place, in contrast with that in pure Cu. The strength of the Cu substrate can be improved by graphene coating, which is explained by the interaction between graphene and dislocations in Cu [79,149]. The strength of the interface increase with the increase in the number of graphene layers [149].

##### **Fe/Graphene** **Composites**

The location of the graphene in the metal matrix as well as its orientation will also affect the mechanical properties of the composite. In Figure 6c, stress–strain curves of the pure iron and the graphene/Fe composite are presented. A pair of single-layer graphene nanoribbons at different locations in the Fe matrix are used as the reinforcement [156]. As can be seen, two graphene nanoribbons parallel to the (11$\bar{2}$) plane increase the strength of Fe considerably. Two main regions are found (OA and AE) with different deformation mechanisms: after point A, dislocation approaches graphene flakes. Graphene reinforcement gives much better strength since, in comparison with intermetallic particles, which are often used as the reinforcement, dislocations cannot easily overcome the graphene plane. Thus, one of the main mechanisms of reinforcement by graphene is the interaction between graphene and dislocations. In the case of Fe/graphene composite, the Orowan “by-passing” mechanism took place, which can be seen in Figure 6c [156]. The dislocation strengthening mechanism is anisotropic and the blocking effect is higher if graphene nanoribbons are oriented normally to the dislocation line.

##### **Ni/Graphene** **Composites**

**Figure 7:**
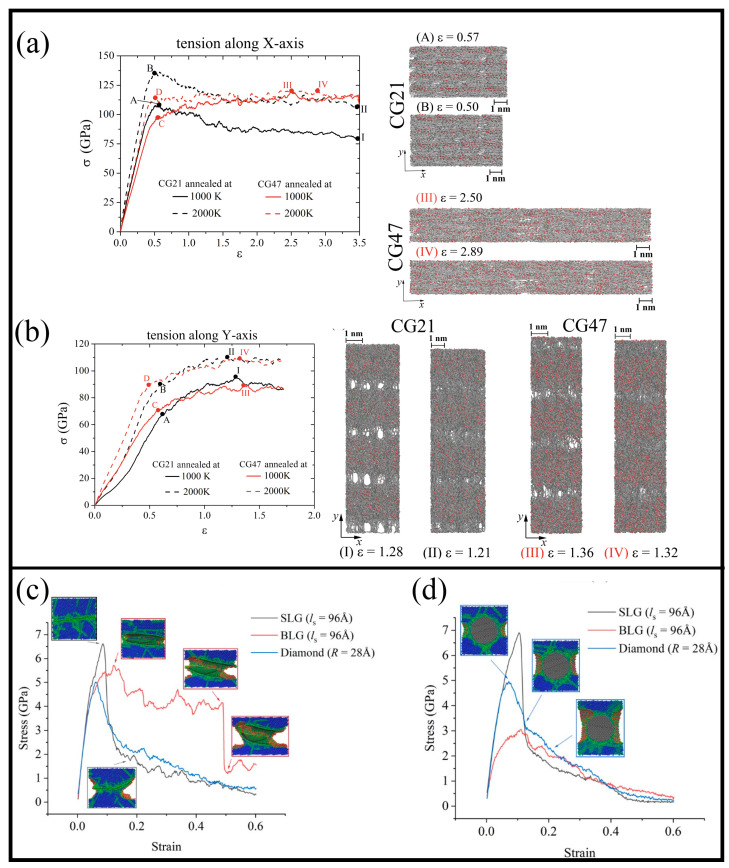
(**a**,**b**) Stress–strain curves for Ni/graphene composite during uniaxial tension along (**a**) *x* and (**b**) *y* with the snapshots of the structure. The composite is crumpled graphene filled with Ni nanoparticles of small (CG21) and average (CG47) size, which was obtained by hydrostatic compression followed by annealing at 1000 K or 2000 K. Reprinted with permission from [18]. (**c**,**d**) The stress–strain curves of sintered Al containing single-layer graphene (SLG), bilayer graphene (BLG), and diamond during tension along (**c**) *y* direction and (**d**) *z* direction. Reprinted with permission from [128].

As shown previously, both isotropic and anisotropic structures can be obtained. In Figure 7, two different composites are presented during tension. In Figure 7a,b, it is a graphene matrix filled with metal nanoparticles, while in Figure 7c,d it is a metal matrix with graphene, bilayer graphene, or diamond sphere as the reinforcing element. In Figure 7a,b, the composite was obtained from crumpled graphene with Ni nanoparticles of small (CG21) and average (CG47) size, which was acquired by hydrostatic compression followed by annealing at 1000 K or 2000 K. The numbers 21 and 47 define the number of Ni atoms inside one graphene flake. The process of fabrication of such composite is presented in Figure 5. The effect of the nanoparticle size for such composites was studied in [17]. It was shown that for composite with a small nanocluster, the composite formation is faster. Temperature increases the velocity of the crumpling process and facilitates the formation of connections between neighboring GFs. Metal nanoclusters of small size are deformed by rigid graphene flake, while bigger nanoparticles stay almost spherical. There is a critical nanoparticle size in accordance with the size of the graphene flake. In the case, presented in Figure 7a,b two more suitable sizes of the nanoparticles are chosen. From the snapshots of the composite at different tensile strains, it can be seen that during uniaxial tension the continuous breaking of bonds and the formation of new ones took place between GFs. The ultimate tensile strength is higher for the composite annealed at 2000 K in case of tension along the *y*-axis (increased by 15% for CG21 and by 23% for CG47).

**Figure 8:**
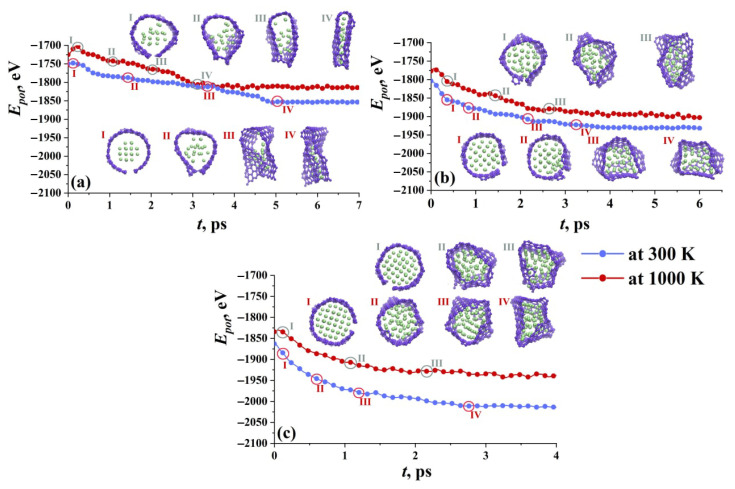
Potential energy as the function of exposure time for graphene flake with different nanoparticles Ni21 (**a**), Ni47 (**b**), and Ni66 (**c**). The snapshots of GF filled with nanoparticles are presented for 300 K (upper snapshots) and 1000 K (lower snapshots). Carbon atoms are shown in violet and nickel atoms are shown in green. Reprinted with permission from [17].

In [17], nanoparticle fillers of different sizes inside the graphene flakes are discussed at different conditions. In Figure 8, three Ni nanoparticles composed of 21 (a), 47 (b), and 66 (c) Ni atoms are presented during exposure at room and finite temperature. The structural state is also characterized by potential energy. The initial ideal shape of the flake and nanoparticle (its f.c.c. crystalline order) starts to change at the very beginning of the exposure for both temperatures. Since there is a strong interaction between Ni and graphene, the nanoparticle is attached to graphene by vdW interaction. For small nanoparticles, graphene flakes can transform into CNT.

For a big nanoparticle, GF just totally covers it, while for a small one, also the crumpling of the flake and changing of its shape took place. At high temperatures, the interaction is much faster, especially for small nanoclusters which can be also melted. In that case, the nanoparticle is divided to separate atoms and spread over the flake. This results in the formation of bi-layer GF with Ni atoms between two layers. For bigger nanoparticles, GFs work as capsules containing metal. However, if the nanoparticle is big, all the free bonds in the basal plane of graphene are occupied, which makes it difficult to form new covalent bonds with other GFs.

In [18] it was revealed that better strength can be observed for the composite with the small nanoparticle since the developed graphene network can be observed for this composite. However, the average size of the nanoparticles also allows the successful fabrication of a graphene/Ni composite. The situation is very different for strong-interacting metals, such as Ni or Pt, and low-interacting metals, such as Cu or Al [157].

**Figure 9:**
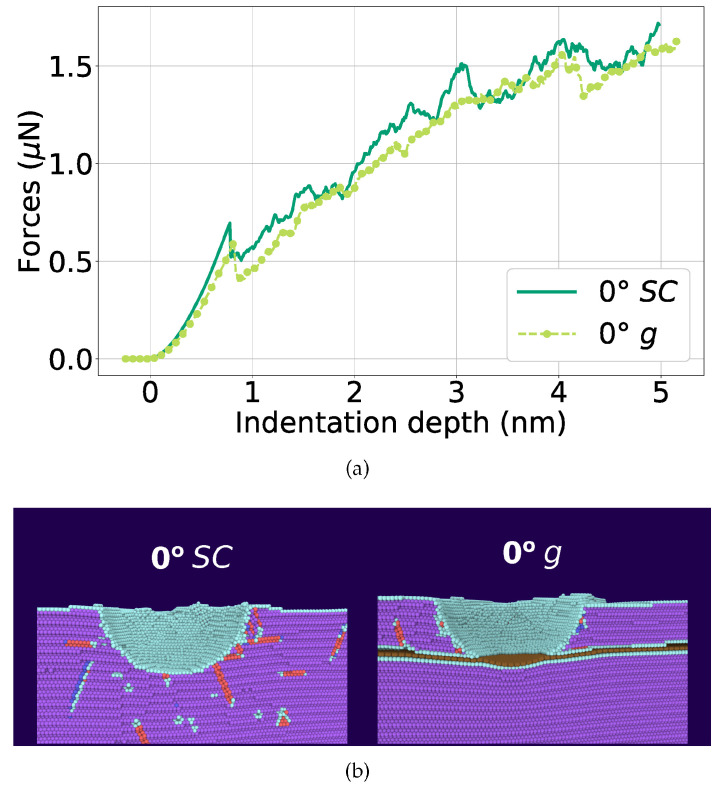
(**a**) Force–indentation depth curve during nanindentation of single-crystalline (SC) Ni (dark-green line) and Ni with graphene (g) (light-green line). (**b**) The snapshots of the structure for pure Ni (SC) and Ni with graphene (g) at an indentation depth of *d* = 4.1 nm, immediately before dislocation nucleation in the lower Ni block. Atoms are colored according to common-neighbor analysis. Purple: fcc; red: stacking faults; cyan: other defects; brown: graphene. Reprinted with permission from [158].

In [158], the nanoindentation of Ni bi-crystal is simulated, where the effect of grain boundary covered with graphene is considered. Commonly, graphene flakes are randomly distributed in the polycrystalline metal matrix. Here, graphene is placed in the interface of two grains with higher and lower misorientation. In Figure 9, the force–indentation depth curve is presented with monocrystalline Ni (SC) and Ni with graphene layer (g) and the snapshots for both structures at a depth of 4.1 nm. The structure is shown before the dislocation nucleation in the lower Ni part. Purple atoms are f.c.c., red are stacking faults, cyan is for other defects, and graphene is brown.

In this case, graphene does not affect the strength of Ni mono- or low-angle Ni bi-crystal. Dislocations are not stopped by graphene. Moreover, graphene bend under indentation and does not increase the composite strength. Moreover, in [71] the dislocations that appeared in the Ni surface covered by graphene never penetrate it but can bend graphene and move inside the Ni part. When an indenter reaches graphene the nucleation of the dislocations below graphene took place which results in cracks formation between Ni and graphene. Thus, in [158], the composite has a smaller hardness than pure Ni. In [159], it was found that under nanoindentation of Ni/graphene composite, its hardness decreases with the increase in the number of graphene layers but at the same time the maximum elastic deformation increases. The strength of the composites can be improved by changing the size and distribution of graphene nanoribbons in Ni/graphene composite, which was shown by nanoindentation [114].

##### **Al/Graphene** **Composites**

In Figure 7c,d, stress–strain curves for tension along the *x*- and *y*-axes are presented for MMC composite reinforced by: (i) graphene; (ii) bilayer graphene, and (iii) diamond sphere. In both cases, the composite containing graphene has the best mechanical properties. In the case of bilayer graphene, there is a difference in tension along different axes, and this composite demonstrates the increase of ductility. It was concluded that a large graphene sheet results in a strength increase, while the addition of bilayer graphene increases the difference in mechanical properties.

Interestingly, this metal type strongly affects the composite behavior under indentation. For example, in [160] the Al/graphene composite is considered with graphene of different sizes. In contrast to Ni, graphene blocks the dislocations, which are piled up near the graphene/Al interface, strengthening the Al matrix. The size of the graphene is of crucial importance: for the larger graphene more dislocations are emitted from the graphene/Al interface if the indenter is far from the graphene. While if the indenter contacts the graphene, the interaction force is lower for a larger graphene sheet due to its high strength.

## 5. Discussion

The following issues should be discussed for a better understanding of the mechanical properties of metal/graphene composites: (1) are these composites anisotropic or isotropic and can the anisotropy be controlled by fabrication technique; (2) how to increase the strength of the composite; (3) how the ratio between some metal and C atoms will affect mechanical properties; (4) deformation and strengthening mechanisms should be defined. The other important issue is to define why and how graphene can increase strength: very different mechanisms were found to date. For example, graphene can participate in the load transfer, and affect the dislocation passing mechanisms and dislocation hardening, resulting in the thermal mismatch between the metallic matrix and graphene. The contribution of such strengthening mechanisms of graphene/metal composites is not always clear.

One of the main parameters affecting the final properties of the graphene/Me composites is their special morphology. In particular, as described in Section 2.1, one can consider a single-layer graphene located in the metal matrix or the whole system of a plane or crumpled graphene layers. As it is known, the strength of graphene is considerably dependent on its chirality. Thus, if the composite is stretched along the graphene plane, we will have a composite with high mechanical properties. Otherwise, if tension is applied normally to graphene, the strength drops sharply, since the bond between the metal and graphene is mainly weak molecular interaction. Moreover, besides, the chirality of graphene is important, as it is known that graphene is stronger when stretched in the zigzag direction and weaker when stretched in the armchair direction. Thus, if graphene is oriented, for example, along the tension direction with a zigzag edge, we will obtain a composite with greater strength. In addition, graphene in a metal matrix can be rotated at a certain angle to the tension axis, this will also change mechanical properties, since the interaction of dislocations with graphene, which affect the strengthening of the composite, will occur in a completely different way. Moreover, free graphene edges act as dislocation sources.

To summarize the reviewed works, we chose such main characteristics important for the simulation of the mechanical properties of the composites as the Me type, type of the potential function, reinforcement, and studied properties. We do not show the obtained values of, for example, ultimate tensile strength or elastic modulus, but just point out what was done to study the mechanical properties. Table 1 provides an overview of the recent (the majority are from the last 5 years) works on graphene/Me composites for Cu, Ni, Al, and Fe. The previous works were discussed and summarized in [3].

The investigation into mono- or polycrystals also affects the mechanical properties of the composite. In the literature, the majority of works with graphene are located in a monocrystalline metal matrix, since this allows us to understand the hardening mechanisms in detail. On the other hand, those works that consider a polycrystalline matrix make it possible to understand how graphene will be introduced into real systems. For example, it is shown that if the grain misorientation is low and graphene is located at the boundary then the strength is lower than with a high-angle grain boundary. In addition, graphene affects the grain structure itself. If we consider the fabrication of a composite, it was found that graphene prevents grain growth; therefore, on the one hand, with graphene, it is possible to obtain a nanocrystalline material in which grain growth is limited, but on the other hand, such a structure may have low strength since graphene is located directly at the grain boundaries.

## 6. Conclusions

This work presents advances in the MD simulation of fabrication and the study of mechanical properties of metal/graphene composites of various morphology. The search for such new composites with improved properties is important in many fields of science and technology. Different aspects of MD simulation studies on the strengthening mechanisms, deformation behaviors, and connections between properties and structures are covered. The present review provides helpful information for future simulation studies on the metal/graphene composite.

It can be concluded that the vdW force between the layers of graphene and some metals is weak, which makes it easy to form interlaminar shear and delamination [111,155,161]. The thickness of the metal layer should be taken into account since, after some critical thickness, the strength of the composite is lower. Several graphene layers also affect the composite properties. If multilayer graphene is chosen as the reinforcement, it can be delaminated during tension, which results in the appearance of voids at the interface between graphene and metal and results in a decrease in the mechanical properties of the composite.

The level of enhancement could be further increased by optimizing the size, volume fraction, and orientation of graphene-based reinforcement materials. One of the perspective ways to further increase strength and mechanical properties is to obtain a graphene network. Moreover, it can be much simpler than reinforcing a metal matrix with a single-layer planar graphene. The understanding of the factors mainly affecting the composite properties can enable one to control the strength of metal/graphene composites by tuning their structure morphologies.

## Figures and Tables

**Table 1:** Overview of MD simulation and properties of graphene-reinforced MMC. NC is for nanocrystalline, Gr is for the graphene layer, NT represents nanotwinned, and GB represents the grain boundary.

Metal	Structure	Potential	Studied Properties	Ref.
Cu	NC Cu matrix with 1 to 4 Gr (embedded)	LJ	friction, shear resistance	[7]
	NT Cu, 1 to 4 Gr (embedded)	LJ	tension, compression, shear	[8]
	Gr with different boundary conditions (embedded),	LJ	nanoindentation, compression, dislocation dynamics	[108]
	NC Cu, Gr, along GBs, from 9.1 vol% to 17.7 vol.%	LJ	tension, dislocation dynamics	[81]
	Gr, CNT (embedded)	LJ	tension, temperature dependence on Young’s modulus	[74]
	1 Gr (embedded)	LJ	shock response	[85]
	1 to 6 Gr (embedded)	LJ	radiation damage resistance	[90]
	6 Gr (on the surface)	LJ	surface cracking	[91]
	NT Cu, 1 to 5 Gr (embedded)	LJ	tension	[92]
	1 to 5 Gr (embedded)	LJ	solidification of liquid Cu with Gr, tension	[94]
	1-3 Gr, crumpled Gr (embedded)	LJ	tension, Young’s modulus	[99]
	1 Gr (embedded)	LJ	interfaces, dislocation nucleation	[101]
	1 Gr (embedded)	LJ	shear strength, Gr pull-out	[102]
	NC Cu, Gr along GBs, from 6.28 vol% to 17.7 vol.%	LJ	tension	[103]
	1 Gr (embedded)	LJ	shock resistance	[105]
	1 to 4 Gt (embedded)	LJ	tension	[109]
	1 to 9 Gr (embedded)	LJ	compression	[89]
	1 Gr (embedded)	LJ	Gr pull-out	[93]
	1 Gr (on the surface)	COMB3	Gr wrinkling	[143]
	1 to 3 Gr	Finnis and Sinclair	tension	[153]
	1 to 3 Gr, different distribution	LJ	tension	[155]
Ni	1 to 4 Gr (on the surface)	LJ	nanoindentation	[95]
	different distribution of Gr	LJ	nanoindentation	[114]
	Gr distribution in Ni	LJ	tension	[96]
	Gr network	Morse	sintering, tension	[20]
	1 Gr (on the surface)	ReaxFF	bending	[136]
	Gr network	Morse	compression, tension	[157]
	1 Gr, on the grain boundary	LJ	indentation	[158]
Cu, Au, Ag	3 Gr	LJ	tension	[97]
Ni, Cu	1 to 8 Gr	LJ	shock compression	[87]
Al	from 1 Gr to 5 vol.%, different orientation	LJ	tension, elastic modulus	[70]
	1 Gr (embedded)	LJ	Interface optimization, tension	[106]
	1 to 3 Gr	LJ	compression, dislocation dynamics	[110]
	Gr of a different size and chirality	LJ	tension	[111]
	5 Gr (embedded)	LJ	nanoindentation, dislocation dynamics	[160]
	1 Gr (embedded)	Morse	sintering, tension	[128]
	1 Gr, 3 Gr, and Gr with a hole (embedded)	LJ	compression	[147]
Fe	2 Gr, different orientation (embedded)	LJ	tension, dislocation dynamics	[107]
	2 Gr	LJ	dislocation-graphene interaction	[108]
	2 Gr, different orientation (embedded)	embedded atom potential	tension, dislocation dynamics	[156]

## Abbreviations

The following abbreviations are used in this manuscript:

MMCs	metal matrix composites
CNTs	carbon nanotubes
2D	two-dimensional
3D	three-dimensional
rGO	reduced graphene oxide
MD	molecular dynamics
CG	crumpled graphene
vdW	van der Waals
LJ	Lennard–Jones
f.c.c.	face-centered cubic
h.c.p.	hexagonal close packed
